# A Comparison of Different Approaches to Quantify Nitric Oxide Release from NO-Releasing Materials in Relevant Biological Media

**DOI:** 10.3390/molecules25112580

**Published:** 2020-06-02

**Authors:** Rosana V. Pinto, Fernando Antunes, João Pires, Ana Silva-Herdade, Moisés L. Pinto

**Affiliations:** 1 CERENA. Departamento de Engenharia Química, Instituto Superior Técnico, Universidade de Lisboa, 1049-001 Lisboa, Portugal; rosana.pinto@tecnico.ulisboa.pt; 2 Centro de Química Estrutural, Faculdade de Ciências, Universidade de Lisboa, 1749-016 Lisboa, Portugal; fantunes@fc.ul.pt (F.A.); jpsilva@fc.ul.pt (J.P.); 3 Instituto de Bioquímica, Instituto de Medicina Molecular, Faculdade de Medicina, Universidade de Lisboa, 1649-028 Lisboa, Portugal; anarmsilva@medicina.ulisboa.pt

**Keywords:** nitric oxide, quantification, biological media, porous materials, oxyhaemoglobin assay, Griess assay, electrochemical sensor

## Abstract

The development of solid materials that deliver nitric oxide (NO) are of interest for several therapeutic applications. Nevertheless, due to NO’s reactive nature, rapid diffusion and short half-life, reporting their NO delivery characteristics is rather complex. The full knowledge of this parameter is fundamental to discuss the therapeutic utility of these materials, and thus, the NO quantification strategy must be carefully considered according to the NO-releasing scaffold type, to the expected NO-releasing amounts and to the medium of quantification. In this work, we explore and discuss three different ways of quantifying the release of NO in different biological fluids: haemoglobin assay, Griess assay and NO electrochemical detection. For these measurements, different porous materials, namely zeolites and titanosilicates were used as models for NO-releasing platforms. The oxyhaemoglobin assay offers great sensitivity (nanomolar levels), but it is only possible to monitor the NO release while oxyhaemoglobin is not fully converted. On the other hand, Griess assay has low sensitivity in complex biological media, namely in blood, and interferences with media make NO measurements questionable. Nevertheless, this method can measure micromolar amounts of NO and may be useful for an initial screening for long-term release performance. The electrochemical sensor enabled real-time measurements in a variety of biological settings. However, measured NO is critically low in oxygenated and complex media, giving transient signals, which makes long-term quantification impossible. Despite the disadvantages of each method, the combination of all the results provided a more comprehensive NO release profile for these materials, which will help to determine which formulations are most promising for specific therapeutic applications. This study highlights the importance of using appropriate NO quantification tools to provide accurate reports.

## 1. Introduction

Nitric oxide (NO) is endogenously produced by nitric oxide synthase (NOS) enzymes, acting as a signalling messenger in many physiological processes including neuronal signalling, immune and inflammatory response, cardiovascular homeostasis and wound repair [1]. Its exogenous administration as a therapeutic agent led to an increasing development of effective NO-releasing therapies, particularly macromolecular scaffolds [2].

Zeolites, titanosilicates and metal-organic frameworks (MOFs) are examples of successful carriers capable of storing therapeutic NO amounts [3,4,5]. They release it in its pure form by exposing the material to aqueous biological environments [6]. As in other NO-release strategies, a full control over the NO release kinetics (i.e., NO flux and half-life) and NO payloads (i.e., amount of NO released over time in aqueous and biological media) are key parameters to develop useful therapies because the biological action of NO is manifested via several chemical reactions that strongly depend on its concentration [7]. Thus, we highlight the importance of studying how to measure and report the NO release from the NO-releasing materials, to allow tuning their characteristics to achieve the best application performance. However, given the complexity of NO chemistry, its quantification is complex and its use for pharmacological purposes is yet very limited. This gaseous molecule contains an unpaired electron that makes it very unstable when in contact with air and in biological environments since it reacts with oxygen and free radical species such as thiols, superoxide, lipid peroxyls and metal-containing proteins (e.g., haemoglobin), giving a very short biological half-life ranging from a few seconds to a few minutes [8,9]. Consequently, the use of sensitive and efficient analytical methods to quantify NO is essential.

Although a wide range of NO analytical methods exists, including highly sensitive gas sensors [10,11,12], only few can quantify the gas under liquid biological environments. Besides, their performances are strongly dependent on the NO-releasing scaffold type (e.g., macromolecular particles, films, gels, coatings, etc.), on the expected range of NO amounts to be quantified and on the medium where the measurement/release takes place. Consequently, selecting the adequate analytical method is crucial for an accurate report and to support further development of these classes of materials.

In this work, we apply three of the most common quantification techniques used in liquid environments (oxyhaemoglobin assay, electrochemical NO sensor and Griess assay) to obtain the NO releasing profiles from various porous NO-releasing materials (i.e., zeolites and titanosilicates). We adapted these methods for the quantification of NO released from solid materials since they were originally developed for NO releasing molecules. More precise analytical methods are available, such as chemiluminescence and high-performance liquid chromatography, but these require costly instrumentation and are often difficult to apply to biological assays.

The most commonly reported method for NO quantification is the Griess assay, which quantifies NO indirectly through the spectrophotometric measurement of nitrite (NO_2_^−^), a stable decomposition product derived from NO autoxidation [13]. This method is inexpensive, fast, and commercially available in ready-to-use formats. However, it has a low detection limit (~0.5 µM) being unsuitable to detect quantities below micromolar [14]. Another colorimetric method used to quantify NO, which in turn is more sensitive, works by monitoring the oxidative reaction between NO and oxyhaemoglobin which generates a shift in the absorption bands that is used as a quantitative indicator of NO. This method is also easy to execute, but its quantification is restricted to haemoglobin solutions. Finally, unlike colorimetric methods, the electrochemical sensor allows direct quantification in situ in any biological environment in real-time, being fairly sensitive (0.3–10 nM) and portable [15].

Herein, we discuss the feasibility and accuracy of each method in measuring the released NO by this type of promising NO carrier in different complex biological media (e.g., protein-rich samples) and consider the inherent limitations of each method.

## 2. Results and Discussion

### 2.1. NO Adsorption Capacity of Different Porous Materials

**Figure 1:**
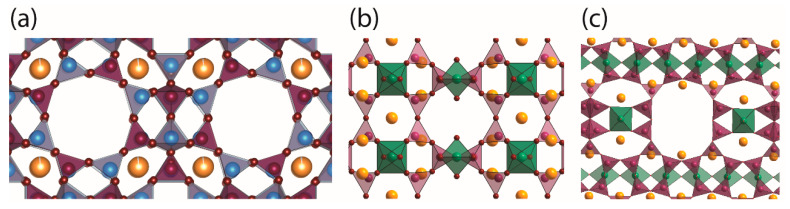
Representation of the pore structure of the materials. (**a**) Zeolite 4A structure with eight-ring pore opening of about 0.4 nm composed of Al (blue) and Si (purple) tetrahedra connected by O, (**b**) ETS-4 structure with eight-ring pore opening of about 0.4 nm composed by Si (purple) tetrahedra and Ti (green) octahedra (one oxygen on the vertical octahedra corresponds to water that can be removed to obtain pentacoordinated Ti) and (**c**) ETS-10 structure with 12-ring pore opening of about 0.8 nm composed by Si (purple) tetrahedra and Ti (green) octahedra. In the case of Co-ETS-4 the extra-framework cations were replaced by Co and in the case of ETAS-10 some Si was replaced by Al, but the structure is analogous to ETS-10. Colour code: Si purple, Ti green, Al blue, O red and cations in yellow. Note: materials labelled ETS are types of titanosilicates.

NO adsorption capability depends on several material characteristics, including the pore and the adsorbed molecule sizes, the cation nature, its distribution and concentration throughout the porous structure, the polarization and the quantity of water [10]. In Figure 1 are depicted the different inorganic porous materials selected for the NO-releasing assessment that had previously shown potential for NO storage [16,17,18,19]. Zeolite A (Figure 1a) contains alternated SiO_4_ and AlO_4_ tetrahedra that share corners to produce the open framework. As can be observed in Figure 1a, the structure has accessible extra-framework cations inside the cavities where NO coordinates through its nitrogen atom, originating a strong interaction by forming either mononitrosyl or dinitrosyl complexes [20]. On the other hand, the titanosilicate ETS-4 (Figure 1b) presents smaller pores that hold unsaturated (pentacoordinate) Ti^4+^ metal centres (one oxygen on the central octahedra pointing to the pore centre corresponds to a water molecule that can be removed) in which NO is coordinated. Also, NO can coordinate to the extra-framework cations that are also accessible in the pores. In contrast with ETS-4, ETS-10 (Figure 1c) Ti^4+^ ions are all hexacoordinated and, consequently, not available for coordination with NO. Thus, NO is expected to bind with the cations inside the channels, as with zeolites. Additionally, modified specimens from ETS-4 and ETS-10 were also studied based on the exchanging extra-framework cations (Na^+^ by Co^+^) (Co-ETS-4) and on the isomorphic substitution of silicon by aluminium (ETAS-10), respectively. In the case of Co-ETS-4, the Co^2+^ provide possibility to a stronger coordination of NO without promoting its degradation [17]. In addition, the exchange of Na^+^ by Co^2+^ increased the pore space available for adsorption, since exchanging a monovalent with a divalent cation leads to a 2:1 exchange ratio. For ETAS-10, the number of surface charges (cations) derived from the framework substitution of the Si^+4^ by Al^3+^ increased, which strongly influenced the NO adsorption and release kinetics.

**Figure 2:**
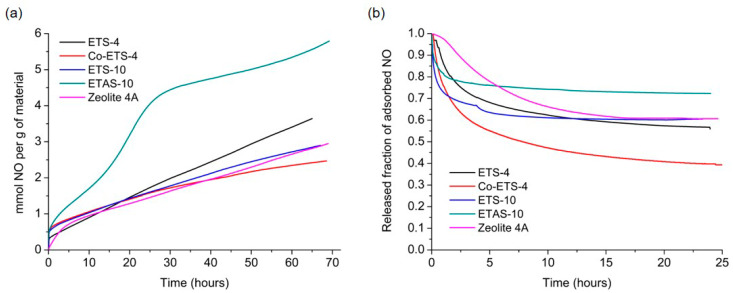
Nitric oxide adsorption and release isotherms at 25 °C on zeolite 4A and on different titanosilicates using a gravimetric apparatus. (**a**) NO adsorption kinetic profiles performed at 80 kPa and (**b**) NO release kinetics in the gas phase using high vacuum. The values released are related to the amount of NO adsorbed by each material. Results from titanosilicates were taken from previous works [17,18].

Gravimetric adsorption and release measurements of these materials are presented in Figure 2a,b, respectively. NO is put in contact with the material at constant pressure (80 kPa) and temperature (25 °C), allowing it to be both physiosorbed and chemisorbed by coordination within the pores. Despite their structural differences and/or different metal sites, the materials presented similar adsorption capacity of ~3 mmol NO per g of material, except for ETAS-10, which exhibits an increased NO loading capacity of 95% when compared with ETS-10, due to the higher cation content on the modified specimen. The accessibility to the pores by gaseous NO is in fact slow due to the narrow pores characteristic of these materials and due to the nature of their chemical surface. Although presenting lower adsorption capacities compared to MOFs, which may reach adsorption capacities of up to ~9 mmol g^−1^ [21], the materials here presented are capable of storing sufficient amounts of NO to provide positive biological effects. Although advantageous, high adsorption capacities should not be the only aspect to be considered in therapeutic applications with NO, since the material should also have the ability to release the stored quantities in a slow and controlled way, in order to guarantee the absence of toxicity in the surrounding tissues.

After NO loading for three days, materials were submitted to NO desorption under high vacuum (Figure 2b). The released amounts are lower than the quantities adsorbed, which is indicative of a strong interaction between NO and the unsaturated metal sites/cations presented into the cavities of the materials during the adsorption stage. Those chemisorbed species cannot be spontaneously released by lowering the NO partial pressure, remaining thus stored inside the frameworks. Hence, this NO can only be released by altering the chemical composition of the material, using, for instance, a suitable nucleophile (water) that replaces the coordination position of NO at the pores surface. Thus, quantification of NO released in the liquid phase is especially important for these materials to draw conclusions more adapted to the reality of the future applications. The quantification in the liquid phase and the comparison of the results from three methods is presented in the following sections, along with a discussion of key experimental issues of each method.

### 2.2. Oxyhaemoglobin Assay

The oxidative reaction between NO and oxyhaemoglobin (oxyHb) (Equation (1)) is considered the main pathway for NO catabolism, being one of the most used mechanisms to analyse NO [22]. The reaction proceeds as follows:

oxyHb + NO→metHb + NO_3_ (1)

**Figure 3:**
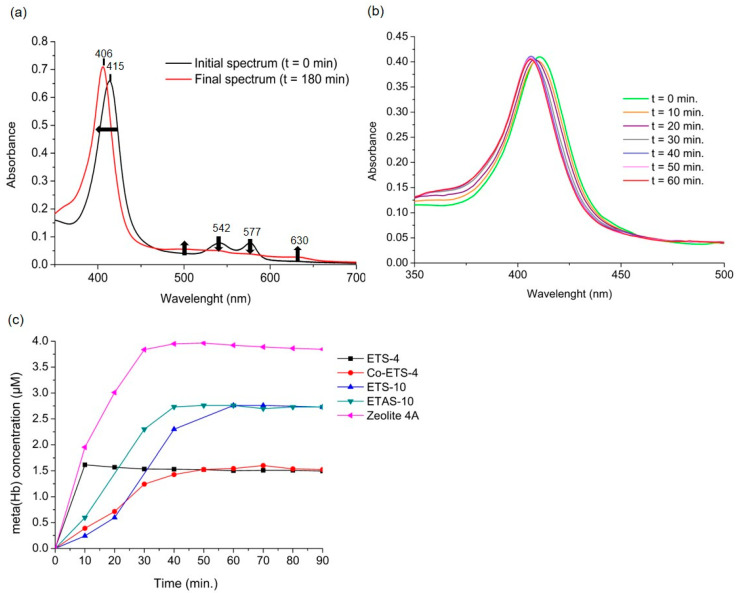
Nitric oxide release profiles in liquid phase from different NO-loaded studied materials using the oxyhaemoglobin assay. (**a**) Comparison between the initial UV/vis spectrum of the oxyhaemoglobin solution and the methaemoglobin spectrum obtained after 180 min in contact with the material (Zeolite 4A). Arrows indicate the change in direction in the absolute spectrum over time. (**b**) Changes over time in the main peak of the oxyhaemoglobin (oxyHb)-containing solution spectrum upon introduction of the NO-loaded material (Co-ETS-4). (**c**) NO release profiles of the different titanosilicates and zeolite 4A obtained at 25 °C in 0.1 M phosphate buffer with 5 µM oxyHb. The concentration of meta(Hb) quantified is considered stoichiometric to the concentration of NO released [23]. Some data from titanosilicates was previously reported [17,18].

This reaction is faster than other competing reactants present in the medium (such as oxygen, superoxide ions, thiols, etc.), which makes the NO capture by oxyhaemoglobin almost stoichiometric in most experimental conditions [23]. Of note, this reaction has been estimated to be 26 times faster than the NO autoxidation, even under high oxygen concentrations, making this method highly sensitive to quantify NO [23]. The oxyhaemoglobin spectrum is depicted in Figure 3a as the initial spectrum before the introduction of the material (t = 0 min). Upon addition of NO, methaemoglobin (metHb) forms resulting in the final spectrum plotted on the same graph. As observed, the NO bonding with the iron present in haemoglobin results in a shift in the most intense peak from 415 nm to 406 nm, as well as a slight variation in the less intense peaks (Figure 3a). The oxidation of oxyHb to metHb is thus monitored by calculating the differences in absorbance from the most intense peak over time, which allows the quantification of NO that is being released by the material (Figure 3b). The slower this reaction is, slower is the NO release by the material since the reaction of the free NO with oxyhaemoglobin is very fast [23].

Following the NO release by the different porous materials (Figure 3c), they all exhibit a rapid release of high NO amounts, since the released NO was able to consume all the oxyHb present in the medium in less than an hour. Since these are powdery materials, samples were ground with poly(tetrafluoroethylene) and mixture was pressed into disks. Then, the disks were loaded with NO and inserted in the cuvette at the beginning of the assay, avoiding thus the scattering of light by the dispersed solid particles through the oxyHb solution that would influence the analysis. ETS-4 and Zeolite-4 were the materials that exhibited the highest release of NO in the shortest period, since after 10 min great amounts of haemoglobin had already been converted to methaemoglobin. Regarding the NO amounts released by each material, they vary according to the porous structure of each material. Despite having similar storage capacities (except ETAS-10), the NO diffusion rate depends on the pores’ size and interconnectivity, as well as the type of bond formed between NO and the structure. With this quantification method it is impossible to have knowledge of the total amount released by these materials, since the quantification measurement ends when the oxyhaemoglobin present in the medium runs out. This means that the material can continue releasing NO, but the medium no longer has oxyHb available and other oxidative reactions can take place that are no longer possible to monitor. For these measurements, the use of as small as possible amounts of this type of material is advisable. For other NO-releasing materials, if its release does not exceed quantities above a few micromolar, this analytical method may guarantee an extended monitoring of their release profiles. According to the protocol described by the authors (Feelisch et al. [23]), the maximum recommended concentration of oxyHb in the solution is 5 µM, since at higher concentrations the absolute absorbance values may be above 2, reducing the accuracy of absorbance readings by several spectrophotometers. In addition, since haemoglobin solutions prepared before each test must pass through a purification process on a Sephadex G-25 column and through an oxygenation process [23], it is impossible to guarantee exactly the same final haemoglobin concentration in all the measurements. Hence, it is impossible to compare the maximum metHb concentration quantified with the material’s ability to release higher or lower NO amounts. Thus, although this method is considered to be very specific and sensitive to NO and capable to detect NO from nanomolar concentrations [24], it is only possible to draw conclusions for these NO-releasing materials regarding their release profiles when the total conversion of oxyHb is verified. In conclusion, the method is highly informative of the initial release rate of NO, which is an extremely relevant information for possible applications. Moreover, we highlight that this method is restricted to use with oxyhaemoglobin solutions as a medium for quantification, and, consequently, to quantify NO in other media, the use of other methods is required (Table 1).

### 2.3. Griess Assay

Griess assay is another colorimetric method used to quantify NO, and relies on a diazotization reaction that was originally described by Griess in 1879 [25]. Briefly, the Griess reaction is a two-step reaction where NO_2_^−^ first reacts with sulphanilamide to yield a diazonium salt intermediate that subsequently reacts with N-1-naphthylethelene diamine to form a chromophoric azo product that can be monitored spectroscopically at 540 nm [26]. The absorbance measured from each solution is then compared with the reference curve, which should use the same matrix or buffer used in the sample to ensure an accurate NO quantification. Since the Griess reaction may interfere with several substances present in the different buffers or matrices, its sensitivity and, consequently, its limit of detection is dependent on each system. According to different sources it can vary from 0.5 to 2.5 μM [27,28], which restricts the application of this method for quantifications to the micromolar range [29]. Additionally, before introduction of the Griess reagent in each sample, care should be taken to remove the NO-releasing material to avoid disturbances in the absorbance measurements. Especially for the materials under study, since these are powdery materials, the analyte solution must be first centrifugated in to separate the materials from the medium. During this period, the material may eventually continue to release NO, thus it is recommended to carry out this process as fast as possible to have better accuracy on the time scale of the release.

In this study, the biological medium was different from the one used for the oxyhaemoglobin assay since Griess reagent cannot be used directly in haemoglobin solutions because it interferes with the spectroscopic chromophoric azo product signal. Thus, complex biological media were chosen as analyte solutions, starting with RPMI-1640 supplemented with foetal bovine serum (FBS), a current medium that supports a wide range of mammalian cells, since the therapeutic potential of the new NO-releasing materials usually is first assessed through in vitro tests.

**Figure 4:**
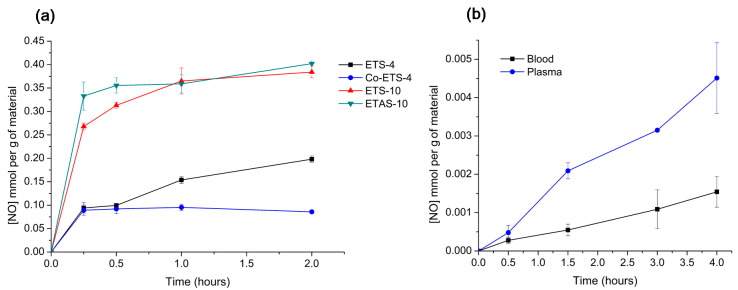
Nitric oxide release kinetics obtained using an indirect measurement through nitrite quantification by Griess assay. (**a**) Release profiles of ETS-4, ETAS-10 and modified specimens at a concentration of 450 µg mL^−1^ in supplemented RPMI-1640 medium at 37 °C [5]. (**b**) Release profiles of Zeolite-4A (11.7 mg mL^−1^) obtained in different biological media (human blood and plasma) at room temperature. *n* = 3; mean ± standard deviation shown.

In Figure 4a, it is possible to compare the NO release profiles of the different titanosilicates obtained through this indirect quantification method. Since, with the amounts of material used, the release of NO is higher than micromolar, this method is useful for studying the total quantities released. According to the adsorption profiles (Figure 2a) within titanosilicate materials, the one that presents the highest NO storage capacity is ETAS-10. However, no significant differences between ETAS-10 and ETS-10 in terms of the total quantities released were observed with the Griess method, which highlights the importance of confirming the release in liquid phase. Both ETS-10 and ETAS-10 revealed a high release (> 60 µM of NO_2_^−^) in the first 15 min, with most of NO being released during that period. This burst of release may cause an adverse biological impact on the cellular activity but can be efficient in the inhibition of the bacterial growth by using the right concentration these NO donors [30,31]. Regarding ETS-4-type materials, both presented lower release of total NO amounts than ETS-10 despite having similar NO storage capacities. These two zeolite-type silicates have distinct porous structures: in contrast with ETS-10, ETS-4 contains pentacoordinate Ti^4+^, which admits a different NO coordination possibility within the structure. These bonds created between NO and unsaturated metal sites are strong, confirmed by the slower release amounts quantified by ETS-type materials.

ETS-4 exhibits a controlled release over time as shown by the slow increase of nitrite in the solution, which is the most favourable release kinetic for drug delivery applications [32]. For the Co-exchanged ETS-4 material, most NO is released in the first few minutes, reaching a final NO concentration of 0.1 mmol g^−1^ after 2 h, considerably less than ETS-4. In this case, the addition of the extra-framework cation Co^2+^ favoured the NO adsorption due to the strong affinity between Co^+2^ and NO, and, consequently, lower NO amounts were released, indicating that the diffusion of NO through the pores is strongly influenced by the chemical composition of the materials surface.

Unfortunately, the total NO release from the quantity of the material deposited is below the maximum NO amounts adsorbed by each material (~3 mmol g^−1^). This could be because, beyond the irreversibly adsorbed NO amounts, the NO detection with the Griess assay in complex media is compromised by the presence of proteins and other additives (e.g., cysteine, tyrosine, ascorbate, and NADPH) [33,34]. The same can be confirmed in Figure 4b, which shows the release from the same material (Zeolite 4A) in different complex media (i.e., blood and plasma). Due to the opacity of the blood and the presence of haemoglobin that is also detected at 540 nm [35], prior incubation with Griess reagent samples were haemolysed. Plasma samples were mixed with NaCl solution to ensure the same dilution performed on blood samples during haemolysis in order to facilitate further comparison of nitrite amounts. In the blood medium, which is the most complex media tested, the total NO measured has been reduced to more than half, comparing the detected NO amounts in plasma due to the expected reaction of NO with blood proteins including oxyhaemoglobin [36]. For quantification in these media, NO-loaded zeolite was used in a disk format (as in the oxyhaemoglobin assay) instead of powder to allow its removal, since centrifugation of blood would separate the plasma from the haematocrit.

Given these results, measuring NO via the Griess method in biological samples is suitable for an initial screening of the NO release behaviour of each material, but the total NO amounts measured in complex media are questionable. However, we stress the importance of measuring NO in relevant milieu according to the desired application before drawing conclusions regarding the therapeutic potential. For quantitative work, this data should be confirmed and supplemented with more accurate analytical measurements. 

In addition, short-term quantification is not possible in the case of these powered materials since the analyte solution must pass through centrifugation prior to the reaction with Griess reagent, which takes at least 5 min. Nevertheless, prolonged NO release profiles can be obtained by measuring the increase of nitrite in the medium. This is not possible neither with oxyhaemoglobin assay nor with electrochemical sensor as shown below (Table 1).

### 2.4. NO Electrochemical Sensor

Electrochemical direct measurements allow obtaining the NO release profiles in almost any biological configuration in real time, representing a significant advantage over the Griess reagent and the haemoglobin assay. The electrode is introduced into the biological solution where the NO donor will be added and the concentration of NO released is quantified by comparing the measured current with the calibration curve, which should be obtained frequently, ideally before each analysis, to assure the data integrity.

The electrochemical detection of NO is fast (seconds) and very sensitive, reaching detection limits as low as 83 pM [37]. However, the more complex the biological setting for quantification is, the bigger the decrease in the response and sensitivity may be verified [34]. Nevertheless, modifying the working electrode by using a catalyst and/or permselective polymer membranes for electrode coatings can be an alternative to increase the NO sensitivity and its selectivity over interfering species [11,34]. 

**Figure 5:**
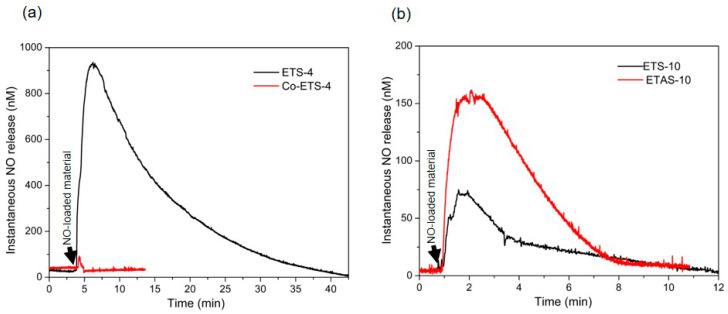
Nitric oxide release profiles from NO-loaded titanosilicates obtained by a direct measurement with NO electrochemical sensor. (**a**) Release profiles of ETS-4 and Co-ETS-4 obtained in supplemented RPMI-1640 medium at room temperature using a materials concentration of 450 µg mL^−1^; (**b**) release profiles of ETS-10 and ETAS-10 obtained in the same conditions as (**a**) but using a different material’s concentration (900 µg mL^−1^).

In this study, we also use the RPMI-1640 medium supplemented with FBS, a complex medium full of proteins. All the titanosilicate materials here presented loaded with NO were tested at 450 µg mL^−1^ to obtain a significant signal, but the released NO could only be measured for ETS-4 and Co-exchanged ETS-4 materials (Figure 5a). Only by increasing the concentration of ETS-10 and ETAS-10 to 900 µg mL^−1^ was it possible to detect measurable NO amounts (Figure 5b).

With this sensor, the maximum NO concentration measured was of 930 nM after 3 min for ETS-4, which is low compared with the NO measured values with the other methods. Unfortunately, the electrochemical method has poor sensitivity in this solution due to the NO reaction with sulfhydryl-containing proteins, such as albumin, macroglobulin and glycoproteins, presented in high concentrations in the FBS supplement added to the medium [38,39]. In addition, the measurements were conducted in the presence of oxygen in order to mimic the biological environment for cell culture, which also poses additional difficulty for NO quantification due to the fast NO oxidation. A previous study developed by Hunter et al. [34] compared the accuracy of measuring NO in a number of oxygenated biological media (such as physiological buffers, cell culture media, urine, saliva and blood) with a NO sensor, revealing that PBS and physiosol were the solutions resulting in the greatest total NO detected (~1.07 µmol mg^−1^), whereas the NO measured in biological fluids containing FBS and blood was significantly lower (~12 × 10^−4^ µmol mg^−1^ in blood). The authors also compared the quantification in oxygenated and nonoxygenated biological media, and, as expected, the total NO detectable increased substantially (~2.4 µmol mg^−1^ in PBS) in nonoxygenated media. 

Unfortunately, these measurements are unable to record long-period release profiles, which limits the total characterization of NO donors. However, useful information may be deduced by observing the duration of active NO released into the biological environment that can be compared with further therapeutic responses, a feature that is impossible to accomplish with the other analytical methods presented here (Table 1).

## 3. Materials and Methods

### 3.1. NO-Releasing Porous Materials and Their Synthesis

Zeolites and titanosilicates are inorganic crystalline materials that own metal ions within their pore structures which allow NO to coordinate [6]. Besides, these may feature numerous architectures by engineering and manipulating their pore network by changing, for instance, the metal site or tuning the pore and particle size, giving different NO binding strengths and diffusion, and consequently different release kinetics.

In this work, we used as a NO-releasing material model a well-known microporous zeolite 4A [Na_86_[(AlO_2_)_86_·(SiO_2_)_106_]·H_2_O] (Sigma-Aldrich, Missouri, MO, USA) [41] and different titanosilicates, in particular ETS-4 [Na_9_Si_12_Ti_5_O_38_(OH)·H_2_O], ETS-10 [(Na, K)_2_Si_5_TiO_13_)·H_2_O] and one modified specimen of each, namely Co-ETS-4 based on the exchanging extra-framework cations (Na^+^ by Co^+^) and ETAS-10, an isomorphic substitution of silicon by aluminium. Titanosilicates were prepared in previous works where their NO storage-release was studied [16,17,18]. Briefly, ETS-4 was prepared by mixing an alkaline solution composed of 33.16 g of metasilicate, 2.00 g NaOH and 3.00 g KCl into 25.40 g H_2_O (mass percentages of 52.2%, 3.1%, 4.7% and 40.0%, respectively). Then 31.88 g of TiCl_3_ (15% *w*/*w*, TiCl_3_ and 10% *w*/*w* HCl) was added to the solution. The resulting gel was placed in a Teflon-lined autoclave and heated at 230 °C for 17 h. The final product was filtered, washed with distilled water and dried at 70 °C overnight. A more detailed procedure was described in previous works [42]. For obtaining Co-ETS-4, 0.5 g of washed ETS-4 was introduced in 50 cm^3^ of CoNO_3_ (0.02 M) and kept under stirring at 50 °C overnight. After centrifuging, the powder was put in contact with CoNO_3_ solution two more times for 2 h and the resulting powder was washed three times with H_2_O [17].

Synthesis of ETS-10 was synthesized as follows: 10.96 g of sodium silicate solution was mixed with 1 g of sodium hydroxide, 1.72 g of potassium fluoride, 6.03 g of titanium trichloride, 0.96 g of sodium chloride and 10 g H_2_O (mass percentages of 35.7%, 3.3%, 5.6%, 19.7%, 3.1% and 32.6%, respectively). Seed crystals (0.1 g) were added to the obtained gel and the crystallization was carried out in an autoclave under autogenous pressure at 200 °C for 64 h. Obtained crystals were further washed and dried at atmospheric temperature. The detailed procedure is described elsewhere [43]. The hydrothermal synthesis of ETAS-10 was carried out under autogenous pressure without stirring, using a typical synthesis gel and a titanium trichloride as Ti source [44].

### 3.2. Nitric Oxide Adsorption/Desorption Isotherms

Kinetic studies of adsorption and desorption of NO were measured using a gravimetric adsorption system composed with a microbalance (C.I. Instruments, Disbal) associated with a high vacuum pump system. The sample (~50 mg) was initially outgassed at either 250 °C (zeolite) or 100 °C (titanosilicates) in vacuum, which was better than 10^−2^ Pa, for 3 h. Furthermore, the temperature of the sample was controlled at 25 °C using a water bath (Grant, GD120) with 0.05 °C precision. The adsorption isotherm was recorded by introducing NO gas (Air Liquide, 99.99%) into the microbalance until reaching 80 kPa of pressure and maintained in contact for three days. The desorption isotherm was conducted by evacuating the NO present in the balance under high-vacuum conditions in order to record the mass loss from the material for more 24 h.

### 3.3. NO Loading

The NO loading was proceeded by introducing the material in a glass vacuum cell which was connected to the vacuum pump system. The sample was outgassed and loaded with NO following the same conditions described for NO adsorption/desorption. After the adsorption period, the non-adsorbed NO gas was evacuated, and the cell was further filled with helium up to atmospheric pressure until the material was used. This methodology ensures the safe storage of the loaded material by minimizing the undesired release/reaction with the air components.

### 3.4. Nitric Oxide Release Quantification in Liquid Phase

#### 3.4.1. Oxyhaemoglobin Assay

NO release studies in the liquid phase were conducted using the oxyhaemoglobin assay, previously described by Feelisch et al. [23]. This is a spectroscopic method that monitors the oxidative reaction between NO and oxyhaemoglobin, which produces methaemoglobin and nitrate. This reaction results in a shift of absorbance from 415 nm (oxyHb) to 401 (metHb) and the released NO is quantified by measuring differences in the absorbance [24]. For these experiments, the powder material was pressed into disks to avoid the dispersion problems of the sample in the liquid form. The disks were composed of 75 % of sample and 25 % of poly(tetrafluoroethylene) powder (m/m) and pressed at 5 tons for 30 s. About 5 mg of disk was loaded with NO following the same procedure described above. 

The oxyHb solution was prepared by dissolving 20 mg of lyophilized haemoglobin in 1 mL of buffer solution and sodium dithionite was added to guarantee the total reduction of haemoglobin. The resulting oxyHb solution was purified by passing it over a Sephadex G-25 column. For NO release quantification, the loaded material was inserted in a quartz cuvette with 3 mL of oxyHb solution (1 μM) and its spectra was recorded every 10 min for 3 h using a UV/Vis spectrophotometer (Genesys 10 S, Thermo Scientific).

#### 3.4.2. Griess Assay

The Griess assay measures the conversion of NO to nitrite [12]. A concentration of 450 µg mL^−1^ of material was immersed in supplemented cell culture medium (RPMI-1640) at 25 °C for a period exceeding its NO release. At predetermined time intervals, the sample was centrifuged in order to separate the material from the medium. Thus, the supernatant containing the released NO was incubated with Griess reagent (2% (*w*/*v*) sulphanilamide in 5% (*v*/*v*) phosphoric acid and 0.2% (*w*/*v*) naphthylethylenediamine dihydrochloride) and the nitrite ions present in the medium reacted with the Griess reagent, producing an azo compound of purple colour, which was quantified by the light absorbance at 548 nm wavelength, using a microplate reader (Tecan, A-5082 Sunrise Remote). The total nitrite concentration was determined using a calibration curve prepared with a sodium nitrite and Griess reagent solutions.

For NO quantification in blood and plasma, human blood samples were taken from six donors after informed consent. In each blood-containing tube, 10 IU mL^−1^ of sodium heparin (anticoagulant) were added. For NO quantification in plasma, blood was first centrifuged at 11000 rpm for 10 min in order to separate the plasma. NO-loaded material of (35 mg mL^−1^; zeolite 4A pressed into disks composed by 33.3% of sample and 66.7% of poly(tetrafluoroethylene) powder (m/m)) were added directly in several plasma and blood aliquots designated for each time point. Right after the end of each period, the sample was separated from the material to interrupt the contact and the release. Prior to incubation with Griess reagent, 200 µL of plasma samples were mixed with 1800 µL of NaCl 0.9% (m/V). Blood samples were haemolysed according to the following protocol: 200 µL of blood was mixed with 800 µL of water, following the addition of 500 µL of 0.3 N barium hydroxide and 500 µL of 0.3 N zinc sulphate. The solution was centrifuged at 11000 rpm for 5 min and the supernatant was incubated with the Griess reagent and the nitrite concentration was determined using the same conditions described above. 

#### 3.4.3. Electrochemical Sensor

NO release profiles were recorded using a direct measurement with a selective NO electrode (ISO-NOP from World Precision Instruments, WPI) previously polarized and calibrated according to the manufacturer’s instructions and measured in supplemented cell culture medium (RPMI-1640 with 10% (*v*/*v*) foetal bovine serum, penicillin-streptomycin (100 UI mL^−1^ and 100 μg mL^−1^, respectively) and 2 mM glutamine). The current output (nA) over time was recorded using the DataTrax2 software. In each measurement, the electrode was inserted in the medium under stirring at room temperature, and a given amount of NO-loaded material was added directly to the medium, reaching a final concentration of 450 µg mL^−1^. The NO released by the samples over time was determined using the daily calibration curve prepared as follows: a solution of 0.1 M H_2_SO_4_ + 0.1 M Kl was prepared and put in contact with the sensor under stirring. Further, different amounts of 50 µM KNO_2_ were successively added and the correspondent current output (nA) was recorded, creating a standard curve.

## 4. Conclusions

Quantification of NO in biological environments is challenging and sometimes the use of more than one analytical method for quantification is important. Data presented here demonstrate significant variations in the quantified NO between different analytical methods. With the oxyhaemoglobin assay (restricted to oxyhaemoglobin solutions), the released amounts are only possible to quantify from the materials with high sensitivity and selectivity until the total conversion of oxyHb in the solution, which is ideal for materials that release nanomolar levels of NO. Regarding the other colorimetric assay based on the Griess reagent, this, in turn, has a lower sensitivity as the quantification medium becomes more complex (e.g., plasma, blood and cell medium). Still, since for instance the materials here studied release high NO amounts (> micromolar), this method is useful to perform an initial screening about the long-term NO release performance. In contrast with both colorimetric methods, electrochemical sensor allows direct measurements under a wide range of biological settings. However, NO quantified electrochemically in biological relevant media cannot be applied to record long period releasing profiles and to evaluate NO-releasing materials with lower capacities, since NO dissolved in the medium is not immediately converted to stable species and can be released from the liquid (as a gas) or react with dissolved oxygen. Table 1 summarizes the most important features of each NO quantification method discussed here.

Despite the drawbacks of each method used, the combination of the results of this study provided a more comprehensive NO release analysis from each material, which will help to determine the most promising formulations according to the desired clinical application. In fact, depending on the desired target application, micromolar or nanomolar concentration ranges can be preferred and a suitable method for each range should be used. For instance, for antibacterial applications that require NO micromolar concentrations, the Griess and haemoglobin assays are more useful, while, for would healing applications, nanomolar therapeutic concentrations are used and thus haemoglobin or electrochemical methods are more suited. Furthermore, our study highlights the importance of reporting accurate NO-release parameters based on adequate NO quantification tools.

## Figures and Tables

**Table 1:** Summary of the main features of the three NO quantification methods studied.

Method	Sensitivity	NO Quantification Periods	Preferred Range for NO Quantification	Advantages	Disadvantages
Oxyhaemoglobin assay	1.3–2.8 nM [40]	Several hours ^1^	up to 4 µM	Inexpensive; does not require specialized equipment;	Indirect; restricted to oxyhaemoglobin solutions; local measurements are not possible
Griess assay	~0.5 µM [14]	Days	0.5 to 100 µM	Rapid and inexpensive; measures NO in a variety of biological fluids; available in ready-to-use kits	indirect; poor sensitivity; located measurements are not possible
Electrochemical sensor	0.3–10 nM [15]	Minutes ^2^	30 nM to 1 µM ^3^	Direct; real-time quantification; Measures NO in a variety of biological fluids; portable; located measurements are possible	Requires constant calibration; membrane has short life span; sensitive to tip position

^1^: Depending on the total NO released amounts, since the quantification lasts until the total conversion of oxyHb is verified. *^2^:* For complex media. The more complex the quantification medium is, the shorter the NO half-life will be. ^3^: May vary depending on the quantification medium quantification.
